# Buffalo Physicians in the Army and Navy

**Published:** 1861-08

**Authors:** 


### BUFFALO PHYSICIANS IN THE ARMY AND NAVY.

*New York State Volunteers.*

CHARLES H. WILCOX, M. D., Surgeon 21st Regiment.

JOSEPH A. PETERS, M. D., Assistant Surgeon 21st Regiment.

LUCIEN DAMAINVILLE, M. D., Assistant Surgeon, 31st Regiment, under Gen. McDowell.

AARON J. STEELE, M. D., Assistant Surgeon, under Gen. Mansfield.

*United States Army.*

CHARLES K. WINNIE, M. D., Assistant Surgeon, under Gen. McClelland.

*United States Navy.*

SAMUEL D. FLAGG, M. D., Assistant Surgeon, U. S. Naval Hospital, Brooklyn.

NEWTON L. BATES, M. D., Assistant Surgeon. Waiting orders.

WILLIAM HOWELL, M. D., Assistant Surgeon. Waiting orders.

IRA C. WHITEHEAD, M. D., Surgeon, Revenue Cutter "Vixen," cruising.

